# Supplementary material for: Evaluation of a New Culture Protocol for Enhancing Fungal Detection Rates in Respiratory Samples of Cystic Fibrosis Patients
Source: J Fungi (Basel). 2020 Jun 9;6(2):82. doi: 10.3390/jof6020082 (PMC7345163; doi:10.3390/jof6020082)
Supplement: Supplementary file 1 [file jof-06-00082-s001.pdf]

Supplementary Materials

Homemade fungal culture media

Medium B+

Amounts per 1000 ml medium

Weigh	**D(+)Glucose-Monohydrate (Merck 1.08342.1000)**	**16,70 g**
	Yeast extract (Conda lab 1702.00)	30,00 g
	Bacto peptone (BD 211677)	6,80 g
	Bacto Agar (BD 214030)	20,00 g
**Measure**	Milli-Q water	973,5 ml
**Dissolve**	Heat until completely resolved	
**pH**	6.6 (± 0.2)	
**Antibiotics**	Ceftazidime; stock solution 12800 mg/L (final conc 32 mg/L)	2,5 ml
	Chloramphenicol; stock solution 40000 mg/L (final conc 50 mg/L)	1,25 ml
	Colistin; stock solution 8000 mg/L (final conc 24 mg/L)	3,00 ml
	Cotrimoxazole; stock solution 12800 mg/L (final conc 128 mg/L)	5,00 ml
**Filling out**	21 ml per Petri dish	
**Shelf life**	3 months at 2-8°C	
**Quality control**	*See quality control	

Scedosporium selective agar (SceSel+)

Amounts per 1000 ml medium

Weigh	**Malt extract (Duchefa M1327.0100)**	**6.25 g**
	Maltose (Sigma 101962921)	6.25 g
	Mono-potassium-phosphate (Sigma 102146710)	1.25 g
	Yeast extract (Conda lab 1702.00)	1.0 g
	Magnesium sulfate 7 H_2O (Sigma **1.05886**)	0.625 g
	Soy peptone (BD 211906)	0.625 g
	Dichloran * One milliliter of a 0.2% solution in ethanol (Sigma 45435)	2 mg
	Benomyl * Dilute in 5 ml ethanol (Sigma 45339)	6 mg
	Bacto Agar (BD 214030)	20.0 g
**Measure**	Milli-Q water	986,5 ml
**Dissolve**	Heat until completely resolved	
**Antibiotics**	Chloramphenicol; stock solution 40000 mg/L (final conc 100 mg/L)	2,5 ml
	Ciprofloxacin; stock solution 40000 mg/L (final conc 100 mg/L)	2,5 ml
	Streptomycin; stock solution 40000 mg/L (final conc 100 mg/L)	2,5 ml
**Filling out**	21 ml per Petri dish	
**Shelf life**	3 months at 2-8°C	
**Quality control**	*See quality control	

Dichloran-Glycerol Agar (DG18)

Amounts per 1000 ml medium

Weigh	DG18 agar base (Oxoid CM0729)	31,5 g
	Glycerol (Duchefa G1345.1000)	220 g
Measure	Milli-Q water	998 ml
Dissolve	Heat until completely resolved	
pH	5,6 (± 0.2)	
Antibiotics	Gentamicin; stock solution 40000 mg/L (final conc 40 mg/L)<br>Chloramphenicol; stock solution 40000 mg/L (final conc 50 mg/L)	1 ml<br>1,25 ml
Filling out	21 ml per Petri dish	
Shelf life	2 months at 2-8°C	
Quality control	*See quality control	

Stock solution antibiotics

Amount (purified)		Measure (incl %loss)	Dissolve in	Final concentration (mg/L)
Ceftazidime	128 mg	128 mg	10 ml DMSO	12800
Ciprofloxacine	400 mg	408,2 mg	10 ml 1 M NaOH	40000
Chloramphenicol	400 mg	408,2 mg	10 ml Ethanol	40000
Cotrimoxazol (1:19 ratio)				12800
Trimethoprim	12,8 mg	13 mg	10 ml DMSO	640
Sulfamethoxazol	243,2 mg	243,2 mg		121600
Colistine	80 mg	81,2 mg	10 ml milli-Q	8000
Gentamicin sulfate	400 mg	428 mg	10 ml milli-Q	40000
Streptomycin sulfate	400 mg	422 mg	10 ml milli-Q	40000

Quality control

Fungus	Origin
Candida krusei	ATCC 6258
Trichosporon spp.	UKNeq 9480
Exophiala dermatitidis	CBS 150.90
Aspergillus fumigatus	ATCC 204305
Aspergillus flavus	ATCC 204304
Aspergillus niger	ATCC 10535
Aspergillus terreus	UKNeq 3748/2812
Penicllium spp.	Personal collection*
Rasamsonia argillacea	Personal collection*
Paecilomyces variotti	ATCC 22319
Scedosporium apiospermum	UKNeq 4104/3841
Lomentospora prolificans	CBS 467.74
Lichtheimia ramosa	B64137
Mucor circinelloides	Personal collection*
Cladosporium spp	UKNeq 9836

* Identification confirmed by sequencing.

Table S1. Detection rate and prevalence of molds in sputum samples of CF patients.

	Year 1				Year 2				Year 3				Year 4			
	Detection rate		Prevalence		Detection rate		Prevalence		Detection rate		Prevalence		Detection rate		Prevalence	
	**n**	**%**	**n**	**%**	**n**	**%**	**n**	**%**	**n**	**%**	**n**	**%**	**n**	**%**	**n**	**%**
Acremonium spp.	0	0%	0	0%	0	0%	0	0%	0	0%	0	0%	2	0.8%	2	2.6%
Alternaria spp.	0	0%	0	0%	0	0%	0	0%	0	0%	0	0%	5	1.9%	5	6.5%
Aspergillus flavus	5	1.6%	3	4.0%	8	2.6%	4	4.9%	7	2.8%	5	6.0%	10	3.8%	6	7.8%
Aspergillus fumigatus	98	31.6%	37	49.3%	105	34.7%	42	51.9%	90	35.4%	39	46.4%	146	55.9%	54	70.1%
Aspergillus glaucus group	2	0.6%	2	2.7%	1	0.3%	1	1.2%	1	0.4%	1	1.2%	3	1.1%	3	3.9%
Aspergillus nidulans	1	0.3%	1	1.3%	0	0%	0	0%	0	0%	0	0%	4	1.5%	4	5.2%
Aspergillus niger	1	0.3%	1	1.3%	2	0.7%	2	2.5%	2	0.8%	2	2.4%	8	3.1%	8	10.4%
Aspergillus ochraceus	0	0%	0	0%	0	0%	0	0%	1	0.4%	1	1.2%	1	0.4%	1	1.3%
Aspergillus spp.	1	0.3%	1	1.3%	3	1.0%	3	3.7%	1	0.4%	1	1.2%	6	2.3%	5	6.5%
Aspergillus versicolor	0	0%	0	0%	0	0%	0	0%	1	0.4%	1	1.2%	1	0.4%	1	1.3%
Aureobasidium pullulans	1	0.3%	1	1.3%	0	0%	0	0%	0	0%	0	0%	0	0%	0	0%
Basidiomycete	4	1.3%	4	5.3%	1	0.3%	1	1.2%	2	0.8%	1	1.2%	7	2.7%	5	6.5%
Cladosporium spp.	0	0%	0	0%	0	0%	0	0%	0	0%	0	0%	6	2.3%	4	5.2%
Colletotrichum spp.	0	0%	0	0%	0	0%	0	0%	0	0%	0	0%	1	0.4%	1	1.3%
Exophiala spp.	0	0%	0	0%	0	0%	0	0%	1	0.4%	1	1.2%	4	1.5%	2	2.6%
Lomentospora prolificans	0	0%	0	0%	0	0%	0	0%	0	0%	0	0%	2	0.8%	1	1.3%
Mucor spp.	0	0%	0	0%	0	0%	0	0%	0	0%	0	0%	1	0.4%	1	1.3%
Paecilomyces spp.	0	0%	0	0%	1	0.3%	1	1.2%	0	0%	0	0%	1	0.4%	1	1.3%
Paecilomyces variotii	0	0%	0	0%	0	0%	0	0%	0	0%	0	0%	1	0.4%	1	1.3%
Penicillium spp.	8	2.6%	7	9.3%	18	5.9%	14	17.3%	18	7.1%	15	17.9%	60	23.0%	36	46.8%
Phoma spp.	0	0%	0	0%	0	0%	0	0%	0	0%	0	0%	1	0.4%	1	1.3%
Purpreocillium lilacinum	0	0%	0	0%	0	0%	0	0%	0	0%	0	0%	1	0.4%	1	1.3%
Rhizomucor pusillus	0	0%	0	0%	0	0%	0	0%	0	0%	0	0%	2	0.8%	2	2.6%
Rhizomucor spp.	0	0%	0	0%	0	0%	0	0%	0	0%	0	0%	1	0.4%	1	1.3%
Rhizopus arrhizus	1	0.3%	1	1.3%	0	0%	0	0%	0	0%	0	0%	0	0%	0	0%
Scedosporium apiospermum	0	0%	0	0%	5	1.7%	1	1.2%	3	1.2%	1	1.2%	1	0.4%	1	1.3%
Scopulariopsis brevicaulis	0	0%	0	0%	1	0.3%	1	1.2%	0	0%	0	0%	0	0%	0	0%
Scopulariopsis spp.	0	0%	0	0%	3	1.0%	1	1.2%	0	0%	0	0%	0	0%	0	0%
Unidentified fungus	0	0%	0	0%	4	1.3%	3	3.7%	5	2.0%	5	6.0%	16	6.1%	14	18.2%
Zygomycete	0	0%	0	0%	0	0%	0	0%	0	0%	0	0%	1	0.4%	1	1.3%
**Any mold**	**114**	**36.8%**	**47**	**62.7%**	**135**	**44.6%**	**54**	**66.7%**	**115**	**45.3%**	**48**	**57.1%**	**198**	**75.9%**	**68**	**88.3%**

Table S2. Detection rate of molds in sputum samples of CF patients that were included in both the retrospective and prospective cohort.

	Rate of detection	
	**Retrospective cohort (n=753)**	**Prospective cohort (n=257)**
Any mold	42.4%	75.5%*
A. fumigatus	35.6%	55.3%*
Aspergillus species	4.1%	12.8%*
Penicillium species	4.8%	23%*
Exophiala species	0.1%	1.6%*
Scedosporium species[a]	0%	1.2%*
Other fungi[b]	2.4%	15.2%*
Any mold excluding *A. fumigatus*	11%	42.0%*

[a] Includes both *Scedosporium apiospermum* and *Lomentospora prolificans*. [b] All fungi that do not group in any of the above. * Significant increase ($p<0.05$) between the prospective cohort and the retrospective cohort.

Table S3. Time until detection.

	Time until detection					
	**Week 1**		**Week 2**		**Week 3**	
	**no. of strains**	**Percentage**	**no. of strains**	**Percentage**	**no. of strains**	**Percentage**
**Time until detection all media**						
Any mold	398	74.3%	105	19.6%	33	6.2%
A. fumigatus	259	79.7%	52	16%	14	4.3%
Aspergillus species	28	70%	12	30%	0	0%
Penicillium species	81	73.6%	22	20%	7	6.4%
Exophiala species	2	40%	3	60%	0	0%
Scedosporium species[a]	2	40%	2	40%	1	20%
Basdiomycetes	0	0%	3	37.5%	5	62.5%
Other fungi[b]	26	60.5%	11	25.6%	6	14%
Any mold excluding *A. fumigatus*	139	65.9%	53	25.1%	19	9%
**Time until detection Sabouraud**						
Any mold	164	85.4%	24	12.5%	4	2.1%
A. fumigatus	119	91.5%	9	6.9%	2	1.5%
Aspergillus species	11	84.6%	2	15.4%	0	0%
Penicillium species	25	75.8%	7	21.2%	1	3%
Exophiala species	2	100%	0	0%	0	0%
Scedosporium species[a]	1	100%	0	0%	0	0%
Basdiomycetes	0	0%	2	100%	0	0%
Other fungi[b]	6	54.5%	4	36.4%	1	9.1%
Any mold excluding *A. fumigatus*	45	72.6%	15	24.2%	2	3.2%
**Time until detection Medium B+**						
Any mold	80	54.8%	50	34.2%	16	11%
A. fumigatus	57	60%	31	32.6%	7	7.4%
Aspergillus species	5	45.5%	6	54.5%	0	0%
Penicillium species	13	52%	9	36%	3	12%
Exophiala species	0	0%	2	100%	0	0%
Scedosporium species[a]	0	0%	0	0%	1	100%
Basdiomycetes	0	0%	0	0%	2	100%
Other fungi[b]	5	50%	2	20%	3	30%
Any mold excluding *A. fumigatus*	23	45.1%	19	37.3%	9	17.6%
**Time until detection Dichloran-Glycerol Agar**						
Any mold	149	77.6%	30	15.6%	13	6.8%

A. fumigatus	81	82.7%	12	12.2%	5	5.1%
Aspergillus species	12	75%	4	25%	0	0%
Penicillium species	43	82.7%	6	11.5%	3	5.8%
Exophiala species	0	0%	1	100%	0	0%
Scedosporium species[a]	0	0%	1	100%	0	0%
Basdiomycetes	0	0%	1	25%	3	75%
Other fungi[b]	13	65%	5	25%	2	10%
Any mold excluding *A. fumigatus*	68	72.3%	18	19.1%	8	8.5%

[a] Includes both *Scedosporium apiospermum* and *Lomentospora prolificans.* [b] All fungi that do not group in any of the above.
